# Cerebral autoregulation across the menstrual cycle in eumenorrheic women

**DOI:** 10.14814/phy2.15287

**Published:** 2022-05-06

**Authors:** Stephanie Korad, Toby Mündel, Jui‐Lin Fan, Blake G. Perry

**Affiliations:** ^1^ School of Health Sciences Massey University Wellington New Zealand; ^2^ School of Sport, Exercise and Nutrition Massey University Palmerston North New Zealand; ^3^ Department of Physiology Faculty of Medical and Health Sciences Manaaki Manawa The Centre for Heart Research University of Auckland Auckland New Zealand

**Keywords:** cerebral autoregulation, eumenorrheic women, menstrual cycle

## Abstract

There is emerging evidence that ovarian hormones play a significant role in the lower stroke incidence observed in pre‐menopausal women compared with men. However, the role of ovarian hormones in cerebrovascular regulation remains to be elucidated. We examined the blood pressure‐cerebral blood flow relationship (cerebral autoregulation) across the menstrual cycle in eumenorrheic women (*n* = 12; mean ± SD: age, 31 ± 7 years). Participants completed sit‐to‐stand and Valsalva maneuvers (VM, mouth pressure of 40 mmHg for 15 s) during the early follicular (EF), late follicular (LF), and mid‐luteal (ML) menstrual cycle phases, confirmed by serum measurement of progesterone and 17β‐estradiol. Middle cerebral artery blood velocity (MCAv), arterial blood pressure and partial pressure of end‐tidal carbon dioxide were measured. Cerebral autoregulation was assessed by transfer function analysis during spontaneous blood pressure oscillations, rate of regulation (RoR) during sit‐to‐stand maneuvers, and Tieck’s autoregulatory index during VM phases II and IV (AI‐II and AI‐IV, respectively). Resting mean MCAv (MCAv_mean_), blood pressure, and cerebral autoregulation were unchanged across the menstrual cycle (all *p* > 0.12). RoR tended to be different (EF, 0.25 ± 0.06; LF; 0.19 ± 0.04; ML, 0.18 ± 0.12 sec^−1^; *p* = 0.07) and demonstrated a negative relationship with 17β‐estradiol (R^2^ = 0.26, *p* = 0.02). No changes in AI‐II (EF, 1.95 ± 1.20; LF, 1.67 ± 0.77 and ML, 1.20 ± 0.55) or AI‐IV (EF, 1.35 ± 0.21; LF, 1.27 ± 0.26 and ML, 1.20 ± 0.2) were observed (*p* = 0.25 and 0.37, respectively). Although, a significant interaction effect (*p* = 0.02) was observed for the VM MCAv_mean_ response. These data indicate that the menstrual cycle has limited impact on cerebrovascular autoregulation, but individual differences should be considered.

## Graphical abstract

We describe cerebral autoregulation in eumenorrheic women in the early and late follicular, and mid‐luteal phases of the menstrual cycle. The rate of regulation following repeated sit‐to‐stand maneuvers was correlated with plasma estrogen, with reduced regulation found in women with greater plasma estrogen. No differences in resting hemodynamic variables were observed across the menstrual cycle. These data highlight the individual variation of the cerebrovascular response to forced oscillations in blood pressure in eumenorrheic women.

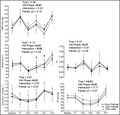

## 1 INTRODUCTION

Men are more likely to suffer a cerebrovascular accident than women, however, this difference diminishes with age (Appelros et al., 2009), with women >55 years. at greater risk than men (Seshadri et al., 2006). The increased stroke risk post‐menopause implicates an effect of the ovarian hormones as it is apparent that estrogen possesses neuroprotective and anti‐inflammatory properties (Spence & Voskuhl, 2012; Vegeto et al., 2008). However, the role of progesterone, particularly during recovery from injury, is less clear (Gibson & Bath, 2016). In addition to its anti‐inflammatory effects, estrogen dilates cerebral vessels, reducing vascular resistance (Belfort et al., 1995; Krause et al., 2006), with middle cerebral artery blood velocity (MCAv), a proxy for cerebral blood flow, reduced in post‐menopausal women (Brislane et al., 2020). The interaction between ovarian hormones is also unclear with progesterone antagonizing the beneficial vascular effects of estrogen (Miner et al., 2011).

Ovarian hormones fluctuate throughout the menstrual cycle in eumenorrheic women, providing a naturally occurring contrast of high and low hormone phases. That is, low estrogen and progesterone during the early follicular (EF) phase, high estrogen only during the late follicular (LF, periovulation) phase, and finally elevations in both estrogen and progesterone during the mid‐luteal (ML) phase. During the LF phase as ovulation approaches (periovulation), plasma 17β‐estradiol peaks, reducing cerebrovascular resistance (Krejza et al., 2001, 2003). The reduction in cerebrovascular resistance during the LF phase of the menstrual cycle mediates a decrease in internal carotid artery (ICA) pulsatility (Krejza et al., 2004), increased ICA blood flow (Nevo et al., 2007), and MCAv (Peltonen et al., 2016). Although, the latter is not a universal finding (Favre & Serrador, 2019). Cote et al (Cote et al., 2021), reported a weak correlation between estrogen and cerebral blood flow, although only compared LF and ML phases and thus not reflecting the full estrogen variability achieved throughout a full menstrual cycle. Therefore, the change in cerebrovascular function across the menstrual cycle has not been completely characterized.

More recently, there has been renewed interest in the potential regulatory role of ovarian hormones on cerebral blood flow, in particular cerebral autoregulation. Cerebral autoregulation is the intrinsic ability of cerebral vessels to buffer fluctuations in perfusion pressure by manipulating cerebral vessel diameter. Whilst there appears to be differences in resting cerebral blood flow between men and women (Ghisleni et al., 2015), few studies have investigated the effect of ovarian hormones on cerebral autoregulation throughout the menstrual cycle (Skinner et al., 2021). Despite animal model data indicating that estrogen modulates myogenic reactivity of cerebral blood vessels (Geary et al., 2000), which is critical to the cerebral autoregulatory response in humans (Tzeng et al., 2011). Assessing cerebral autoregulation using repeated squat stand maneuvers and sit‐to‐stand tests, Favre and Serrador (Favre & Serrador, 2019) found no change across the EF, LF, and ML phases. However, salivary ovarian hormones were measured as a proxy for serum concentrations; with estrogen unchanged across the menstrual cycle and only 8 of the 13 women demonstrating an increase during the LF phase. Addressing the functional outcomes of cerebral blood flow regulation, (Claydon et al., 2006) assessed orthostatic tolerance across the EF, LF, and ML phases of the menstrual cycle with no change observed. Although, similarly to Favre and Serrador (Favre & Serrador, 2019), no change in serum estradiol or autoregulatory indices, determined by the correlation coefficient of the relationship between MCAv and blood pressure, were reported across the menstrual cycle. Others have shown trends for lower MCAv and pulsatility index during a supine‐sit‐stand in the ML compared to EF phase, with no change in the cerebrovascular response to the Valsalva maneuver (VM) (Abidi et al., 2017). However, (Abidi et al., 2017) did not examine the cerebrovascular responses to the VM during the LF phase, and a confounding effect of progesterone cannot be excluded.

Thus, a paucity of data exists exploring cerebral autoregulation and responses to blood pressure challenges during phases of the menstrual cycle when circulating estrogen is confirmed to be elevated. By accounting for variations in menstrual cycle length, EF and LF phases provide a natural physiological variation to assess cerebrovascular function when estrogen is low and elevated respectively, whilst minimizing the potential confounding influence of progesterone. Furthermore, to assess the potential interaction effect of ovarian hormones, and compare responses to existing research investigating the effect of ovarian hormones on cerebrovascular VM responses (Abidi et al., 2017), the ML phase is included in this experiment. The aim of this study was to assess the effect of endogenous ovarian hormones on cerebral autoregulation and cerebrovascular responses at rest and during rapid changes in blood pressure in eumenorrheic women.

## 2 MATERIALS AND METHODS

### 2.1 Ethics and informed consent

An a priori power analysis (G*Power version 3.1.9.4; Heinrich Heine University Düsseldorf, Düsseldorf, Germany) was conducted using data from Abidi et al. (Abidi et al., 2017) with similar interventions (low and high hormone menstrual cycle phases and the VM), design and outcome measures (i.e., MCAv). Based on conventional α (0.05) and β (0.80) values, a minimum of nine participants were required. However, to allow comparisons to data from (Abidi et al., 2017) we decided to recruit twelve women. Twelve healthy eumenorrheic women (self‐reported regular menses for ≥12 months, mean ± SD: age, 31 ± 7 years; height 167 ± 6 cm, weight 74 ± 16 kg) were recruited for the study. All participants were non‐smokers, had no history of amenorrhea, polycystic ovarian syndrome, cardiovascular, pulmonary, metabolic, or neurological disease. Participants were not using any form of hormonal contraceptive (e.g., oral, implantable or intrauterine device for >1 year) and were recreationally active. All participants were informed of the potential risks and experimental procedures, and informed written consent was obtained. All procedures and protocols were approved by the Massey University Human Ethics Committee (SOA 18/77) and performed in accordance with the Declaration of Helsinki.

### 2.2 Study design

Participants visited the temperature‐controlled laboratory four times, once to receive a full familiarization session and the remaining visits comprising the three experimental trials. All equipment and protocols were explained to the participants in the familiarization session as follows. The middle cerebral artery was insonated as described below (see Measurements). Participants then practiced the sit to stand maneuver, and lastly a seated 15 s end‐inspiratory VM at a mouth pressure of 40 mmHg (both described in the experimental protocol). Mouth pressure was measured via transducer and displayed in real‐time to the participant.

A urinary‐based digital fertility monitor that detects both luteinizing hormone (LH) and estrogen (Clearblue Fertility Monitor, SPD Swiss Precision Diagnostics) was provided to participants at the familiarization session. Participants were instructed to use the fertility monitor daily following the cessation of their next menses until peak fertility (LH surge) was indicated. Once peak fertility was identified, participants would record the number of days from the onset of menses (day 1 of cycle) until peak fertility was identified, then fertility monitor use would cease for that cycle. Participants tracked at least two menstrual cycles prior to completion of the experimental trials to inform the timing of data collection.

Participants repeated the experimental protocol three times, representing different phases of the menstrual cycle. (1) Early follicular—conducted on day 3 ± 1 of the menstrual cycle, with day 0 being the onset of menses as confirmed by the participant. (2) Late follicular—timing was approximated from the onset of menses to the onset of peak fertility in previous cycles and confirmed by a fertility monitor on the day of the experiment, which equated to day 13 ± 2 of the menstrual cycle. (3) Mid‐luteal—timing approximated using previous menstrual cycles and was taken as the midpoint between ovulation (estimated by peak fertility indication of fertility monitor) and the estimated onset of menses in the subsequent cycle, equating to day 22 ± 3 of the menstrual cycle.

### 2.3 Experimental protocol

**FIGURE 1:**
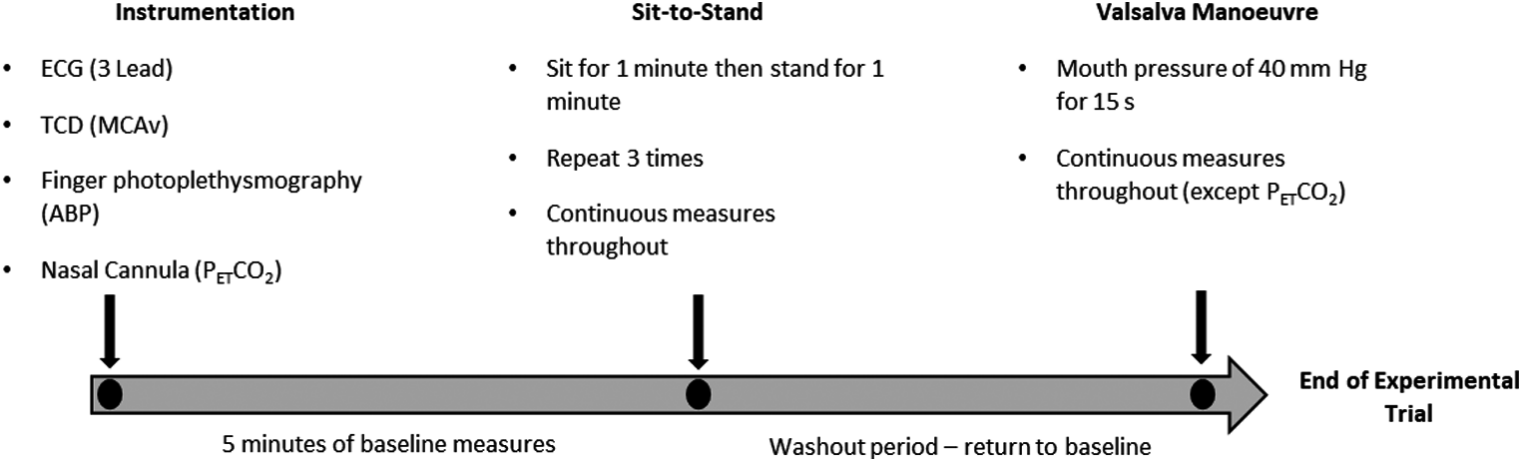
Experimental Protocol. ECG, Electrocardiogram; TCD, transcranial Doppler; MCAv, middle cerebral artery blood velocity; ABP, arterial blood pressure; P_ET_CO_2_, Partial pressure of end‐tidal carbon dioxide

The order of the experimental trials was dependent on participant availability and participant’s position within their menstrual cycle relative to the familiarization session, resulting in some participants involvement in the study spanning multiple menstrual cycles. Irrespective of starting position within the experiment, all trials were separated by >6 days Participants arrived at the laboratory having refrained from caffeinated beverages for 12 h, and vigorous exercise and alcohol consumption for ≥24 h prior to testing. The experimental overview is shown in Figure 1.

Upon arrival, the participants were seated for 20 min for instrumentation. During all experimental conditions heart rate (HR), arterial blood pressure, and middle cerebral artery blood velocity (MCAv) were measured continuously. As recommended (Labrecque et al., 2017), this study utilizes multiple methods for perturbing mean arterial blood pressure (MAP) for the assessment of dynamic cerebral autoregulation. Firstly, following 5 min of seated baseline participants completed sit‐to‐stand maneuvers as previously described (Favre & Serrador, 2019; Labrecque et al., 2019; Sorond et al., 2009). Briefly, from a seated position, participants were instructed to stand quickly (in ≤3 s) and remain standing for 1 min, then sit for 1 min. The process was repeated three times. Participants were instructed to avoid propelling themselves upward by using their hands on their thighs during the standing movement, to breath spontaneously, and to minimize movement once standing.

Following the completion of the sit‐to‐stand maneuvers, participants were seated and remained seated until circulatory measures (HR and MAP) approximated those of the initial baseline period. Following 1 min of normal tidal breathing, participants then performed a seated, end‐inspiratory VM, by blowing into a circular mouthpiece sufficient to generate a mouth pressure of 40 mm Hg for 15 s. As in the familiarization session, visual feedback of strain duration and intensity was provided. A mouth pressure of 40 mm Hg has been used previously to generate significant perturbations in blood pressure to assess cerebral autoregulation (Tiecks et al., 1996; Tiecks, Lam, Matta, et al., 1995) and also allows comparison with existing data (Abidi et al., 2017). Furthermore, a 15s strain enables all phases of the VM to be identified, not possible in strains <10 s (Perry, Mündel, et al., 2014). At strain onset, a rapid increase in MAP ensues as the now elevated intrathoracic pressure is translated to the arterial vasculature (phase I, PI). The elevated intrathoracic pressure impedes atrial filling such that cardiac output, and subsequently MAP, declines (phase IIa, PIIa). Baroreflex mediated increases in HR produce a recovery in MAP (phase IIb, PIIb), followed by an acute reduction in MAP at strain cessation (phase III, PIII). However, PIII reductions in MAP are posture dependent, with limited decreases observed whilst supine (Pott et al., 2000). Finally, a rapid increase in MAP ensues (Phase IV, PIV) due to the combination of restored cardiac output and elevated sympathetic tone persisting from the previous phases (Pott et al., 2000; Tiecks, Lam, Matta, et al., 1995). Once the VM was completed, measurement of partial pressure of end‐tidal carbon dioxide (P_ET_CO_2_) resumed.

### 2.4 Measurements

#### 2.4.1 Cardiorespiratory variables

Heart rate was measured using a three‐lead electrocardiogram (ECG, Lead II; ADInstruments, Australia). Non‐invasive beat‐to‐beat arterial blood pressure was measured by finger photoplethysmography (Finometer MIDI, Finapres Medical Systems, The Netherlands). The cuff was placed on the right hand on either the middle finger or the index finger of the participant and referenced to the level of the heart. The system includes a height correction unit that accounts for hydrostatic pressure differences between the hand and heart, as produced by orthostasis in this experiment. The finometer was corrected against a calibrated automated sphygmomanometer (Sure signs VM4, Philips Medical systems, USA). MCAv was measured using transcranial Doppler ultrasonography (Doppler‐BoxX, DWL, Compumedics, Germany). Blood velocity in the M1 segment of the middle cerebral artery was measured using a 2 MHz probe fixed over the temporal window fixed in position via an adjustable headband. The M1 segment was identified using search techniques described elsewhere (Aaslid et al., 1982; Willie et al., 2011). P_ET_CO_2_ was measured using a breath‐by‐breath online gas analyzer (Model ML206, ADInstruments, Australia) and was collected throughout the experimental trial using a nasal cannula.

#### 2.4.2 Ovarian hormones

The Clearblue Advanced Digital fertility monitor detects both LH and estrogen with high (96%–97%) accuracy (Su et al., 2017), whereby ovulation was correctly predicted within two days of urinary LH peak day in 123 of 135 ovulatory cycles (Behre et al., 2000). Therefore, urinary measurement of LH and estrogen was used to predict ovulation and, therefore, the timing of trials in addition to onset of menstruation. To confirm the menstrual cycle phase, a venous blood sample was collected within 60 min of each trial via venipuncture by a trained phlebotomist at a Southern Community Laboratory. Analysis for 17β‐estradiol and progesterone concentrations were completed on‐site using electrochemiluminescence immunoassay analysis (Roche Cobas e602). Successful ovulation was estimated by ML phase progesterone, with plasma concentrations >9.5 nmol l^−1^ the definition criteria (Hatcher et al., 1988). The progesterone limit was set to exclude anovulatory cycles from the data set. All trials were completed at the same time of day with diet matched between trials.

### 2.5 Data analysis

#### 2.5.1 Calculation of mean values

Mean MCAv (MCAv_mean_) and MAP were calculated as the integral for each cardiac cycle divided by the corresponding pulse interval. An index of cerebrovascular conductance (CVCi) was calculated via the equation MCAv_mean_/MAP. The Gosling pulsatility index (Pi) for MCAv was calculated as systolic MCAv (SMCAv)—diastolic MCAv (DMCAv)/MCAv_mean_. For MCAv_mean_ and MAP the absolute nadir, the absolute change from baseline, and relative change from baseline were determined for each sit‐to‐stand maneuver.

#### 2.5.2 Rate of regulation

The Rate of Regulation (RoR) was calculated to assess the MAP and MCAv_mean_ relationship during acute hypotension following sit‐to‐stand maneuvers. RoR was calculated as previously described (Labrecque et al., 2019) using the equation:

RoR=(ΔCVCi/Δt)/ΔMAP,

where (ΔCVCi/Δ*t*) is the linear regression slope between the change in CVCi and the change in time (*t)* during the initial regulatory response to the sit to stand maneuver (Aaslid et al., 1989; Ogoh et al., 2008). The Δ*t* is a 2.5 s interval following the individually determined initial regulatory response (continual rise in CVCi) upon standing (Labrecque et al., 2019). The change in MAP (ΔMAP) is the difference between the pre‐stand MAP, averaged over 4s immediately preceding the stand (Aaslid et al., 1989), and the average MAP during the initial hemodynamic response to standing where MCAv_mean_ changes occur independent of baroreflex control (Van Beek et al., 2008).

#### 2.5.3 Autoregulatory index

Tieck’s autoregulatory index (AI) method (Tiecks, Lam, Matta, et al., 1995) was used to assess dynamic cerebral autoregulation during Phase II and IV of the VM using the following equations:

Phase II of the VM:

$$\text{AI‐II}=\frac{\text{MCAv}\;\text{(phase}\;\text{IIb‐phase}\;\text{IIa)/MCAv}\;\text{(phase}\;\text{IIa)}}{\text{MAP}\;\text{(phase}\;\text{IIb‐phase}\;\text{IIa)/MAP}\;\text{(phase}\;\text{IIa)}}.$$

Phase IV of the VM:

$$\text{AI‐IV}=\frac{\text{MCAv}\;\text{(phase}\;\text{IV)/MCAv}\;\text{(phase}\;\text{I)}}{\text{MAP}\;\text{(phase}\;\text{IV)/MAP}\;\text{(phase}\;\text{I)}}.$$

Values that are greater than 1.00 indicate that autoregulation is present, while values less than 1.00 indicate that autoregulation is absent (Tiecks, Lam, Matta, et al., 1995).

#### 2.5.4 Power spectral density and transfer function analysis

The assessment of 5 min of spontaneous resting BP and MCAv parameters has been previously described in detail and will be outlined herein (Allan et al., 2015). Raw 1 kHz BP and MCAv waveforms were decimated by a factor of 4 Hz to a sampling rate of 250 Hz, and spline interpolation was performed on mean BP and MCAv using Ensemble (Version 1.0, Elucimed Ltd, Wellington, New Zealand) (Zhang et al., 1998). Data were then passed through a Hanning window and fast Fourier transformed based on the Welch algorithm (50% overlap) in the very low frequency (VLF, 0.02–0.07 Hz), low frequency (LF, 0.07–0.2 Hz), and high frequency (HF, 0.2–0.4 Hz) ranges. These bands have been identified as physiological significance in human (Stauss, 2007), and based on the high‐pass filter characteristics of CA. Linear transfer function analysis was performed on the interpolated BP and MCAv data (window length: 100 s; spectral smoothing: Three point window; coherence threshold: 0.6) (Zhang et al., 1998), which provide three interpretable parameters that reflect the linearity (coherence), magnitude (gain), and the timing (phase) relationships between BP and MCAv as a function of frequency. To account for the inter‐individual variations in middle cerebral artery diameter MCAv spectral power and gain were expressed in normalized units, defined as the signal divided by the mean (Allan et al., 2015).

### 2.6 Data acquisition

All data were collected continuously using an analog to digital converter (PowerLab, ADInstruments, Australia) interfaced with a computer and then analyzed using LabChart software (v8.1.12 ADInstruments, Australia).

### 2.7 Statistical analysis

All data were analyzed using SPSS statistical software version 26 (IBM Corp., Armonk, NY, USA), with statistical significance set at *p* ≤ 0.05. A one‐way analysis of variance (ANOVA) was performed to compare baseline measures, RoR, AI, post VM and the average absolute and relative MAP and MCAv values at Nadir following the sit‐to‐stand maneuvers across the experimental trials (EF, LF, and ML). A repeated measures two‐way ANOVA was used to analyze dependent variables of interest throughout the VM for all conditions (PI, PIIa, PIIb, PIII and PIV × EF, LF, and ML). Post‐hoc pairwise comparisons were used to isolate main effects in the data (Bonferroni corrected). Effect sizes were estimated using partial eta squared (partial η^2^, interaction effect only with two‐way ANOVA), with large effect sizes identified as >0.14, medium 0.06–0.14, and small <0.06 (Cohen, 2013). Simple linear regression was used to explore relationships between 17β‐estradiol in isolation (EF and LF phases) and cerebral autoregulatory metrics and resting MCAv_mean_. All data are displayed as mean ± SD.

## 3 RESULTS

### 3.1 Participant dropout/compliance

**FIGURE 2:**
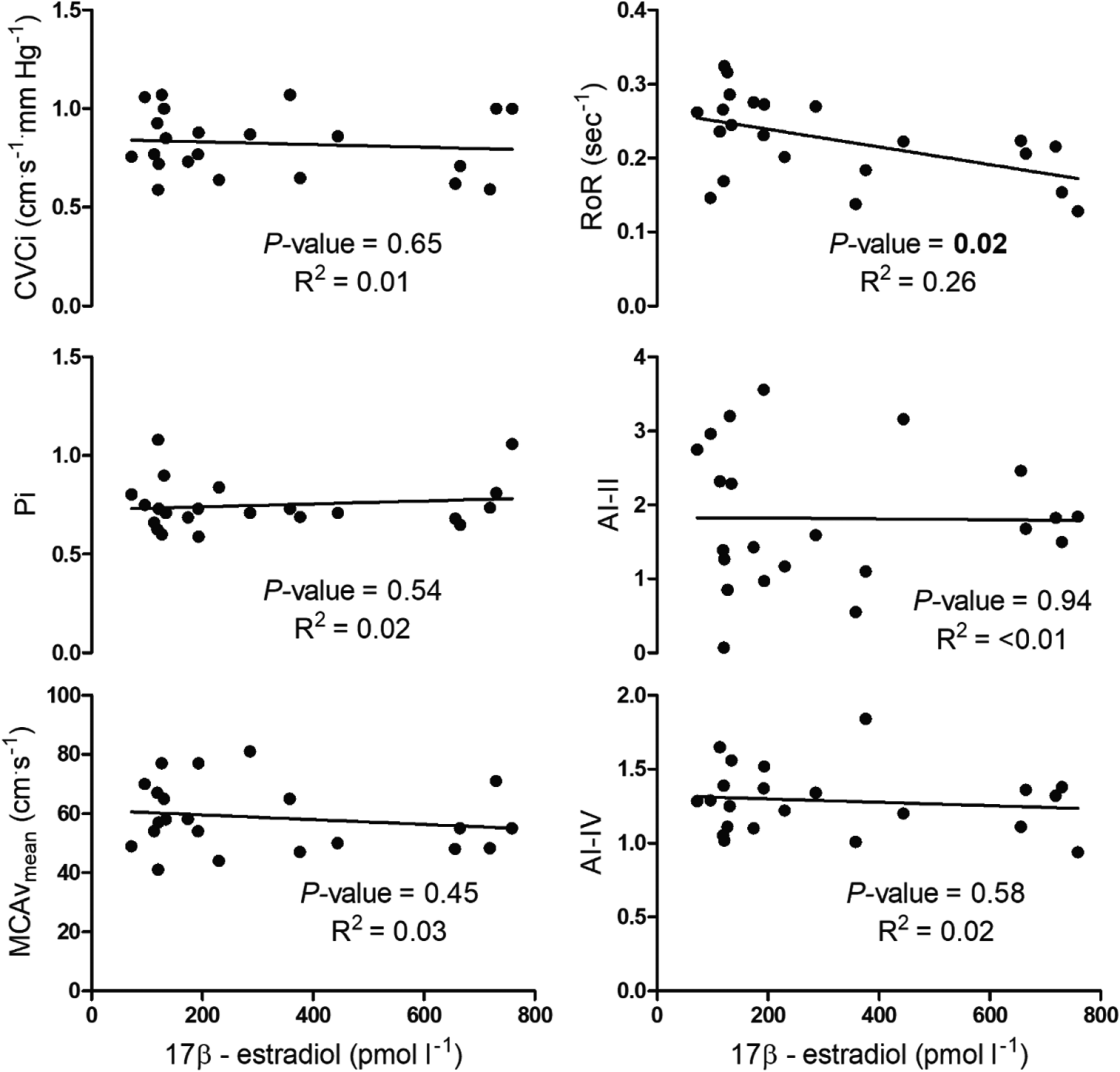
Relationship between resting cerebrovascular conductance index (CVCi), pulsatility index (Pi), mean middle cerebral artery blood velocity (MCAv_mean_), the average rate of regulation (RoR) as calculated following sit‐to‐stand maneuvers, and Tieck’s autoregulatory index (Tiecks, Lam, Matta, et al., 1995) during phase II (AI – II) and phase IV (AI‐IV) of the Valsalva maneuver, and 17β‐estradiol during EF and LF phases only (*n *= 12 for all, 12 × EF and 10 × LF trials)

Two participants withdrew voluntarily before completion of all trials (one participant only completed the EF trial, whilst another completed the EF and ML trials). A third participant did not demonstrate a significant increase in progesterone during the mid‐luteal phase, indicating an anovulatory cycle and/or inappropriate timing of testing. As the purpose of the regression analysis was to investigate the relationship between estrogen and primary outcome measures, only data from EF and LF phases were included. If the participant had completed the EF trial, LF trial, or both, their data were included in the linear regression analysis (Figure 2). Accordingly, only EF and LF data were included in the regression analysis, which included 22 data points (12 × EF and 10 × LF). For the comparison of cerebrovascular function across all phases of the menstrual cycle *n* = 9.

### 3.2 Ovarian hormone concentrations

Concentrations of the primary ovarian hormones are shown in Table 1. Briefly, 17β‐estradiol concentrations were significantly greater in the LF and ML phases than the EF phase (see Table 1 for *p* values). Furthermore, progesterone peaked in the ML phase and was greater than both EF and LF phases of the menstrual cycle. Collectively, these data confirm the correct timing of the menstrual cycle phase and corroborates fertility monitor data.

**TABLE 1:** Ovarian hormone concentrations

	Early follicular	Late follicular	Mid‐luteal	*p*‐value	Partial *η* ^2^
17β – estradiol (pmol l^−1^)	136 ± 31	500 ± 211^a^	424 ± 224^a^	**<0.01**	0.78
Progesterone (nmol l^−1^)	1 ± 1	2 ± 2	29 ± 13^a^, ^b^	**<0.01**	0.54
Progesterone/Estrogen Ratio	10 ± 2	6 ± 6	76 ± 47^a^, ^b^	**<0.01**	0.89

Note

Data are means ± SD. The bold values indicate significant *p*‐values.

^a^ Significantly different from early follicular (*p* ≤ 0.015).

^b^ Significantly different from late follicular *(p* ≤ 0.01*)*.

### 3.3 Baseline values

Baseline cerebrovascular and cardiorespiratory measures are shown in Table 2 below. No differences in any resting values were observed between trials (all *p* > 0.16). In addition, resting values in the baseline period immediately preceding the VM were not different between trials (all *p* > 0.20).

**TABLE 2:** Participant’s resting cerebrovascular and cardiovascular measurements

	Early follicular	Late follicular	Mid‐luteal	*p*‐value	Partial *η* ^2^
MCAv_mean_ (cm.s^−1^)	62 ± 12	57 ± 13	62 ± 11	0.25	0.16
MAP (mm Hg)	72 ± 7	67 ± 8	68 ± 13	0.16	0.21
HR (bpm)	70 ± 16	72 ± 12	73 ± 13	0.47	0.09
CVCi (cm.s^−1^. mm Hg^−1^)	0.86 ± 0.16	0.88 ± 0.24	0.88 ± 0.22	0.85	0.02
Pi	0.75 ± 0.15	0.76 ± 0.13	0.77 ± 0.13	0.91	0.03
PP (mm Hg)	64 ± 3	64 ± 14	66 ± 6	0.46	0.20
P_ET_CO_2_ (mm Hg)	41 ± 3	40 ± 3	40 ± 4	0.53	0.08

Note

Data are means ± SD.

Abbreviations: CVCi, cerebrovascular conductance index; HR, heart rate; MAP, mean arterial blood pressure; MCAv_mean_, mean middle cerebral artery blood velocity; P_ET_CO_2_, partial pressure of end‐tidal carbon dioxide; Pi, pulsatility index; PP, pulse pressure.

### 3.4 Cerebral autoregulation at rest

Spectral and transfer function analysis of spontaneous fluctuations in blood pressure and MCAv at rest are shown in Table 3. No changes in any metrics were observed across the menstrual cycle (all *p* > 0.12).

**TABLE 3:** Spectral and transfer function analysis of spontaneous fluctuations in blood pressure and MCAv at rest

	Early follicular	Late follicular	Mid‐luteal	*p*‐value	Partial *η* ^2^
ABP Total Power (mm Hg^2^.kHz^−1^)	20.2 ± 16.4	23.7 ± 16.9	16.8 ± 13.8	0.51	0.09
MCAv Total Power ((cm.s^−1^)^2^.kHz^−1^)	16.8 ± 12.3	20.7 ± 17.5	19.0 ± 15.2	0.60	0.07
High Frequency (0.20–0.40 Hz)					
ABP Power (mm Hg^2^.kHz^−1^)	3.0 ± 3.7	1.2 ± 0.9	1.8 ± 1.8	0.25	0.18
MCAv Power ((cm.s^−1^)^2^.kHz^−1^)	2.4 ± 2.0	1.2 ± 0.7	2.5 ± 1.6	0.12	0.26
Coherence (AU)	0.72 ± 0.18	0.61 ± 0.19	0.64 ± 0.15	0.13	0.23
Gain (cm.s^−1^.mm Hg^−1^)	1.06 ± 0.40	0.95 ± 0.29	1.18 ± 0.56	0.36	0.12
nGain (%.mm Hg^−1^)	1.74 ± 0.53	1.70 ± 0.61	1.95 ± 0.83	0.62	0.06
Phase (radians)	0.12 ± 0.26	−0.12 ± 0.38	0.33 ± 0.61	0.12	0.23
Low Frequency (0.07–0.20 Hz)					
ABP Power (mm Hg^2^.kHz^−1^)	10.9 ± 11.8	14.7 ± 17.3	8.8 ± 8.6	0.43	0.12
MCAv Power ((cm.s^−1^)^2^.kHz^−1^)	9.2 ± 8.3	14.5 ± 17.5	10.4 ± 12.0	0.38	0.13
Coherence (AU)	0.74 ± 0.14	0.73 ± 0.12	0.76 ± 0.09	0.85	0.02
Gain (cm.s^−1^.mm Hg^−1^)	1.01 ± 0.30	0.96 ± 0.21	1.04 ± 0.25	0.60	0.06
nGain (%.mm Hg^−1^)	1.67 ± 0.36	1.68 ± 0.37	1.71 ± 0.22	0.88	0.06
Phase (radians)	0.34 ± 0.31	0.36 ± 0.15	0.59 ± 0.42	0.14	0.22
Very Low Frequency (0.02 – 0.07 Hz)					
ABP Power (mm Hg^2^.kHz^−1^)	8.2 ± 5.2	9.5 ± 6.2	7.2 ± 5.5	0.79	0.03
MCAv Power ((cm.s^−1^)^2^.kHz^−1^)	7.0 ± 5.1	6.9 ± 3.9	7.3 ± 4.8	0.93	0.01
Coherence (AU)	0.48 ± 0.23	0.42 ± 0.11	0.44 ± 0.12	0.76	0.03
Gain (cm.s^−1^.mm Hg^−1^)	0.85 ± 0.44	0.97 ± 0.53	0.99 ± 0.31	0.58	0.24
nGain (%.mm Hg^−1^)	1.34 ± 0.53	1.64 ± 0.69	1.64 ± 0.43	0.57	0.25
Phase (radians)	0.71 ± 0.47	0.86 ± 0.67	0.87 ± 0.56	0.72	0.15

Note

Data are means ± SD.

Abbreviations: MAP, mean arterial blood pressure; MCAv, middle cerebral artery blood velocity; nGain, normalized gain.

### 3.5 Cerebrovascular and cardiovascular response to repeated sit‐to‐stand maneuvers

There were trends for the absolute change in MCAv_mean_ immediately following the sit‐to‐stand to be different across the menstrual cycle despite no differences in MAP (see Table 4 for values). Whilst only a trend for RoR to be different across the menstrual cycle phases, correlating serum 17β‐estradiol and RoR revealed a significantly negative relationship (see Figure 2).

**TABLE 4:** Autoregulatory and hemodynamic responses to sit‐to‐stand maneuvers

	Early follicular	Late follicular	Mid‐luteal	*p*‐value	Partial *η* ^2^
RoR (sec^−1^)	0.25 ± 0.06	0.19 ± 0.04	0.18 ± 0.12	0.07	0.28
MCAv_mean_ nadir (cm.s^−1^)	50 ± 12	47 ± 12	48 ± 11	0.43	0.10
Δ MCAv_mean_ at nadir (cm.s^−1^)	−12 ± 5	−11 ± 3	−13 ± 7	0.07	0.28
Δ MCAv_mean_ at nadir (%)	−18 ± 9	−20 ± 7	−21 ± 9	0.21	0.18
MAP nadir (mm Hg)	50 ± 11	49 ± 11	50 ± 15	0.95	0.01
Δ MAP at nadir (mm Hg)	−25 ± 9	−24 ± 6	−26 ± 10	0.87	0.02
Δ MAP at nadir (%)	−32 ± 11	−32 ± 11	−33 ± 17	0.94	0.01

Note

Data are means ± SD across the three performed sit‐to‐stand maneuvers.

Abbreviations: MAP, mean arterial blood pressure; MCAv_mean_, mean middle cerebral artery blood velocity; Rate of regulation; RoR.

### 3.6 Cerebrovascular and cardiovascular response to the Valsalva maneuver

**FIGURE 3:**
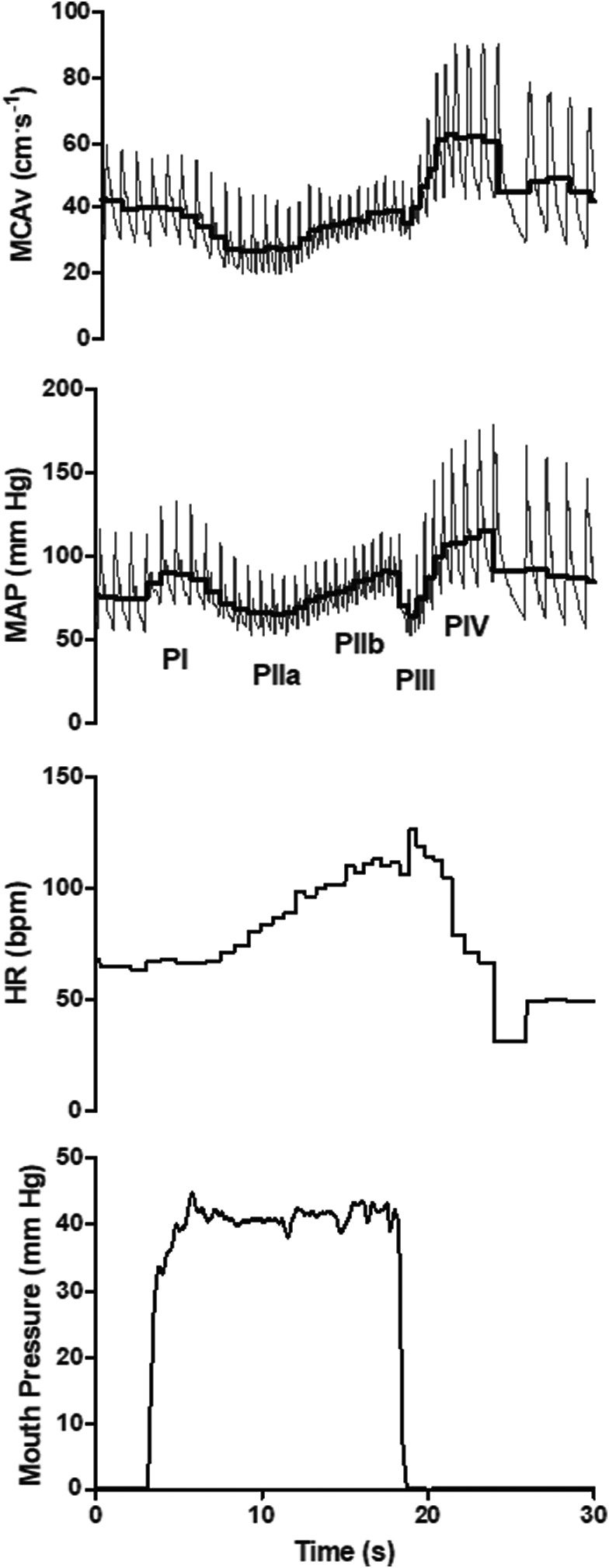
Typical trace for a 40 mm Hg Valsalva maneuver. MCAv, middle cerebral artery blood velocity; MAP, mean arterial blood pressure; HR, heart rate, and mouth pressure as a surrogate for intrathoracic pressure; PI, Valsalva maneuver phase I; PIIa, phase IIa; PIIb, phase IIb; PIII, phase III; PIV, phase IV. Thick black line represents the mean value for each cardiac cycle within the MAP and MCAv trace

**FIGURE 4:**
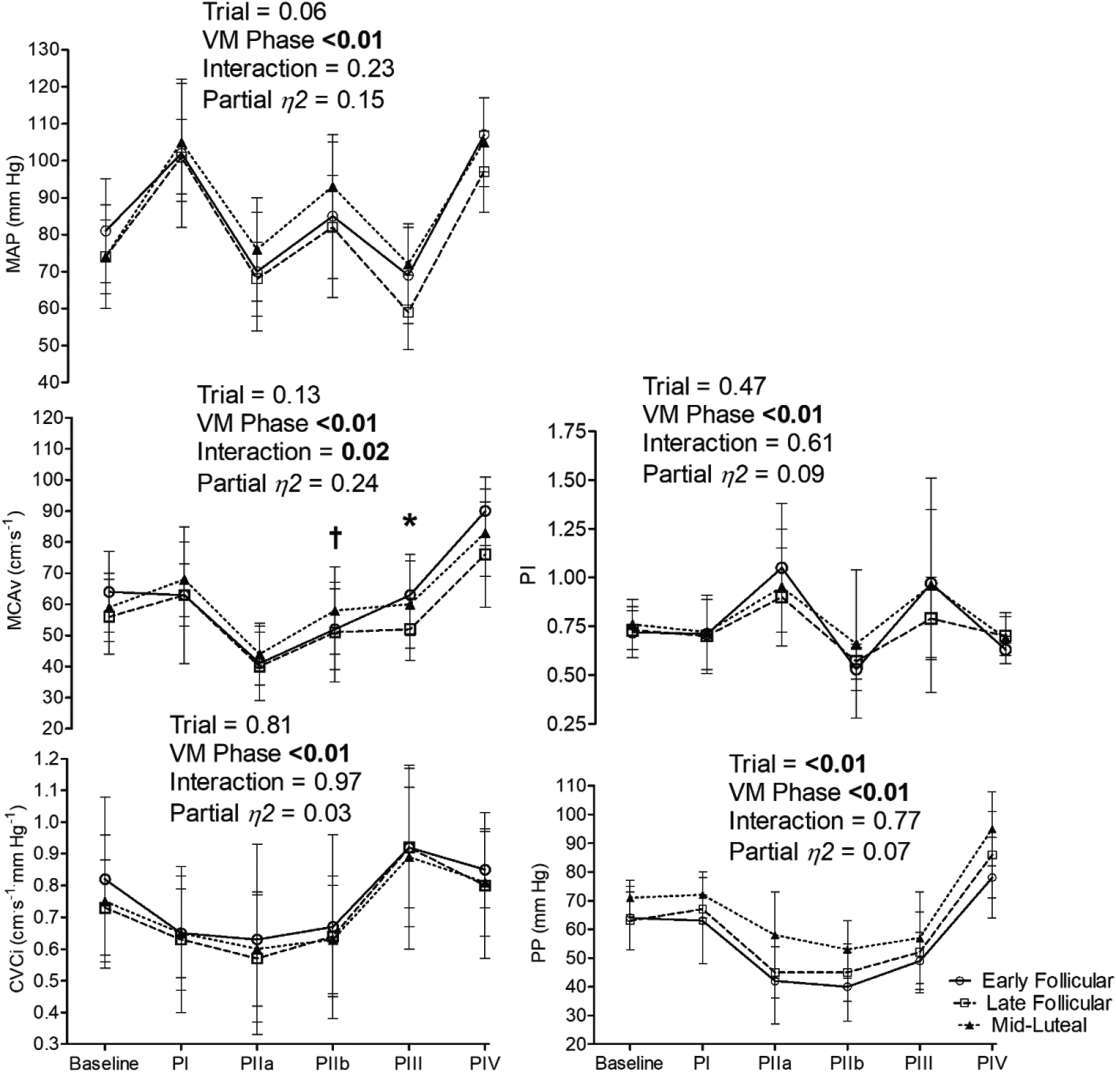
Valsalva maneuver hemodynamics across the menstrual cycle. MAP, mean arterial blood pressure; MCAv_mean_, mean middle cerebral artery blood velocity; CVCi, cerebrovascular conductance index; Pi, pulsatility index; PP, pulse pressure; PI, Valsalva maneuver phase I; PIIa, phase IIa; PIIb, phase IIb; PIII, phase III; PIV, phase IV. *, significant difference between late follicular and mid‐luteal within Valsalva maneuver phase (*p *= 0.02) and trend for the difference between late follicular and early follicular (*p *= 0.06); †, significant difference between early follicular and mid‐luteal within Valsalva maneuver phase (*p *= 0.03). Data are means ± SD

Cerebrovascular and cardiovascular responses to the VM are shown in Figures 2, 3, 4 and Table 5. An interaction effect for MCAv_mean_ was observed (*p *= 0.02, Partial *η* ^2^ = 0.24). Post hoc analysis between menstrual cycle phases revealed significant differences between LF and ML within PIII of the VM (*p *= 0.02, see Figure 4 for values). An interaction effect for SMCAv was also seen (*p *= 0.05, Partial *η* ^2^ = 0.21), although post‐hoc tests comparing data during VM phase in isolation (e.g., PIIa) across the menstrual cycle (e.g., EF vs. LF) revealed no differences (all *p* > 0.05). No difference in VM derived autoregulatory indices were observed (see Table 5). The change in MCAv_mean_ from the previous VM phase was not different across the menstrual cycle phase with pooled means 4 ± 11, −23 ± 11, 12 ± 9, 4 ± 12, and 25 ± 10 cm.s^−1^ for Δ baseline to PI, Δ PI to PIIa, Δ PIIa to PIIb, Δ PIIb to PIII, and Δ PII to PIV respectively (Interaction *p *= 0.39, Partial *η* ^2^ = 0.12). An interaction effect was observed for the percentage change from baseline for MCAv throughout the VM (*p *= 0.04, Partial *η* ^2^ = 0.22), however, post hoc analysis only revealed differences during PIII between EF and LF (2 ± 15 vs. −8 ± 10%, *p *= 0.02). Change from the previous phase or percentage change from baseline for MAP and CVCi were not significant across the menstrual cycle (Interaction all *p* > 0.06). There were no significant differences in the first full breath post VM (EF =39 ± 4 mm Hg, LF =39 ± 4 mm Hg, and ML =38 ± 4 mm Hg, *p *= 0.75, Partial *η* ^2^ = 0.04).

**TABLE 5:** Autoregulation metrics during the Valsalva maneuver

	Early follicular	Late follicular	Mid‐luteal	*p*‐value	Partial *η* ^2^
AI‐II	1.95 ± 1.20	1.67 ± 0.77	1.20 ± 0.55	0.25	0.16
AI‐IV	1.35 ± 0.21	1.27 ± 0.26	1.20 ± 0.24	0.37	0.12

Note

Data are means ± SD.

Abbreviations: AI‐II, autoregulatory index for phase II (initial strain regulation): AI‐IV, autoregulatory index for phase IV (post strain regulation); AI; Autoregulatory Index (AI).

## 4 DISCUSSION

The aim of the current study was to investigate the effect of circulating estrogen on cerebral autoregulation and cerebrovascular responses to rapid perturbations in blood pressure. The primary results indicate that RoR following sit‐to‐stand maneuvers has a weak negative correlation to serum 17β‐estradiol, with only a trend for RoR to be different across the menstrual cycle. Furthermore, MCAv_mean_ during PIII of the VM was different between LF and ML, although VM derived autoregulatory metrics were unchanged. Despite the tendency for cerebrovascular function to differ during sit‐to‐stand induced blood pressure perturbations, transfer function derived metrics of cerebral autoregulation during spontaneous fluctuations in MAP, and resting MCAv and CVCi, were unchanged across the menstrual cycle. Importantly, estrogen and progesterone were significantly different between menstrual cycle phases. Collectively, these data indicate that cerebrovascular regulation during acute changes in blood pressure is largely unchanged throughout the menstrual cycle, although individual variation in estrogen may produce slight differences in regulation following acute reductions in blood pressure.

### 4.1 Resting hemodynamics

No change in transfer function derived autoregulatory metrics during spontaneous blood pressure oscillations were observed across the menstrual cycle (Table 3). Similarly, resting MCAv_mean_, CVCi, Pi, and MAP were not different between menstrual cycle phases (see Table 2) with no relationship observed between 17β‐estradiol and resting hemodynamic variables (Figure 2). Our data indicating no change in resting MCAv_mean_ across the phases of the menstrual cycle aligns with the findings of Favre & Serrador, 2019, but not (Peltonen et al., 2016). Peltonen et al., 2016 reported an increase in resting MCAv_mean_ during the LF phase compared to EF, with data during ML not collected. When comparing blood velocity in the ICA across the menstrual cycle Krejza has reported increases (Krejza et al., 2001, 2004), and no change (Krejza et al., 2013), from EF (Day 3) to LF (Day 13–14 periovulation), with the increases observed in the former studies suggested to result from an estrogen mediated reduction in downstream vessel resistance (Krejza et al., 2003). Similarly, during ovarian stimulation ICA blood flow increased by ~22% in the late follicular phase (Nevo et al., 2007). The reason for these discrepancies is unclear, although comparing resting blood velocities between extra‐ and intracranial vessels may contribute. Using magnetic resonance imaging, cerebral blood flow demonstrated a weak, but positive, correlation with estrogen by Cote et al., 2021. Although, the study by Cote and colleagues only included data from the LF and ML phases thereby not reflecting the full physiological range of circulating estrogen that would be expected within the menstrual cycle (namely low estrogen as in EF). Ghisleni et al., 2015 reported no correlation between cerebral perfusion and estrogen, however, the menstrual cycle phase was not controlled and data were collected at a single time point. Importantly, these studies only reflect resting, steady‐state, function. Recently there has been some debate regarding the appropriateness of assessing cerebral autoregulation at rest during spontaneous MAP oscillations versus purposefully perturbing MAP to challenge the cerebral vasculature (driven oscillations) (Simpson & Claassen, 2018; Tzeng & Panerai, 2018). Whilst there is still some disagreement, assessing both resting hemodynamics and “stressed function” during driven oscillations in MAP (and therefore cerebral perfusion pressure) may elucidate the more specific role of estrogen as discussed in the next section.

### 4.2 Cerebrovascular response to sit‐to‐stand maneuvers

There is some debate whether the menstrual cycle should be controlled in vascular physiology research (Stanhewicz & Wong, 2020; Wenner & Stachenfeld, 2020). Specifically, a paucity of studies has investigated the effect of ovarian hormones on cerebral autoregulation during forced oscillations in blood pressure. Whilst we demonstrate no change in cerebral autoregulation during spontaneous oscillations in blood pressure, we did observe a weak 17β – estradiol dependent relationship with RoR in response to the acute hypotension produced following sit‐to‐stand maneuvers (Figure 2). That is, cerebral autoregulation, as assessed by RoR, became less effective during reductions in blood pressure as circulating 17β‐estradiol increased. These findings add to a growing body of data that supports estrogen as a potential modulator of cerebral blood flow when a stressor is introduced. In alignment with the suggestions of others, reporting individual hormone concentrations has elucidated differences (Skinner et al., 2021). Estrogen may modulate endothelium‐dependent nitric oxide‐mediated vasodilation, as Iwamoto et al., 2021 found shear‐meditated dilation of the ICA during hypercapnia is positively correlated with estradiol, with lower estradiol associated with reduced shear‐mediated dilation independent of age. Furthermore, larger relative increases in estrogen during ovarian stimulation are positively correlated with ICA blood flow (Nevo et al., 2007). In animal models, estrogen modulates myogenic reactivity of cerebral vessels via endothelium‐derived cyclooxygenase (Geary et al., 2000), favoring the production of prostacyclin, thereby stimulating vasodilation (Ospina et al., 2003). In eumenorrheic women the increase in resting MCAv during the LF phase was abolished during cyclooxygenase inhibition with indomethacin (Peltonen et al., 2016), indicating similar modulation may exist in humans. As myogenic tone is implicated in autoregulation (Willie et al., 2014), the modulation of cerebral myogenic reactivity by estrogen may affect autoregulatory capacity, potentially in a dose‐dependent manner. As we, and others (Favre & Serrador, 2019), did not find any change in resting cerebral autoregulatory function during spontaneous fluctuations in blood pressure only forced perturbations may elucidate differences in function across the menstrual cycle. The functional impact of estrogen on cerebral autoregulation is unclear, with orthostatic tolerance unchanged across the menstrual cycle (Claydon et al., 2006; Meendering et al., 2005). Furthermore, how progesterone and estrogen collectively modulate cerebrovascular function is unclear. At rest, progesterone is negatively correlated with blood flow. In the brachial artery progesterone administration abolishes estrogen mediated improvement in flow mediated dilation (Miner et al., 2011) and the progesterone appears to have an opposing effect on cerebral blood flow (Ghisleni et al., 2015), although examining the interactive effect of these hormones is beyond the scope of the current study.

There are several instances throughout the lifespan that provide a naturally occurring contrast in ovarian hormones to assess their effect on cerebrovascular function, one being menopause. Brislane et al. (Brislane et al., 2020) reported that transfer function derived gain during repeated squat‐stand maneuvers was lower in postmenopausal (~59 ± 6 years of age) compared to premenopausal (~33 ± 9 years of age) women. Unexpectedly, a reduced gain in postmenopausal women (low estrogen) would indicate improved autoregulation, although no changes in normalized gain or other autoregulatory parameters were reported. Testing within the premenopausal group was also conducted in the first 7 days of the menstrual cycle (EF), and therefore, represents the low estrogen phase. Another opportunity to investigate the effects of estrogen on cerebrovascular function using a within‐subject design is during endogenous fluctuations in ovarian hormones across the menstrual cycle, as adopted in the current study. To the authors’ knowledge, only one other study has assessed autoregulation across similar phases of the menstrual cycle as the current study (e.g., EF, LF, and ML). Favre and Serrador (Favre & Serrador, 2019) reported no difference in gain or phase during repeated squat stand maneuvers, or Tieck’s (Tiecks, Lam, Aaslid, et al., 1995) autoregulatory index during sit‐to‐stand across the menstrual cycle in the MCA or anterior cerebral artery territories.

Our results corroborate those of Favre and Serrodor as we observed only a trend for dynamic cerebral autoregulation to be different across the menstrual cycle as assessed by RoR. However, comparing data between studies is problematic for several reasons. Firstly, different methods of assessing cerebral autoregulation during forced blood pressure oscillations precludes direct comparison. Secondly, Favre and Serrador (Favre & Serrador, 2019) reported no change in salivary estradiol across the three measured time points of the menstrual cycle (EF, LF, and ML), with only 8 of the 13 participants demonstrating an increase in estradiol from EF to LF. As illustrated in the current experiment, and previously (Lei et al., 2017), timing and targeting of a specific phase of the menstrual cycle is challenging with the length of cycle variable between participants. Whilst the data from one participant was excluded from the within‐subject analysis in the current study, the serum hormone concentrations corroborate the data from menstrual cycle tracking pre‐participation, confirming that testing was conducted in the desired phase.

### 4.3 Cerebrovascular response to the Valsalva maneuver

The Valsalva maneuver produces a complex circulatory response with rapid changes in cardiac output and blood pressure (Pott et al., 2000) that challenge the regulation of cerebral blood flow (Perry, Cotter, et al., 2014). The rapid changes in blood pressure provide an opportunity to assess cerebral autoregulation as first described by Teicks et al. (Tiecks, Lam, Matta, et al., 1995). No differences in the autoregulatory index for PII (AI‐II) or PIV (AI‐IV) were found across the menstrual cycle. Although, we did show minor differences when comparing the percentage change in MCAv_mean_ from baseline with a greater PIII percentage reduction in LF compared with EF. Similarly, when comparing the absolute PIII MCAv_mean_ LF values were lower than ML, and tended to be lower than EF. These data corroborate our results in the sit‐to‐stand in that it appears more likely that differences in cerebrovascular regulation across the menstrual cycle occur during forced hypotensive challenges.

Whilst simple to perform, the cerebrovascular response to the VM is confounded by varying intracranial pressure (Haykowsky et al., 2003). The rise in intracranial pressure impacts vessel transmural pressure, subsequently increasing the critical closing pressure during the strain and confounding the quantification of cerebral autoregulation (Dawson et al., 1999). However, even when critical closing pressure would be expected to be rapidly reducing as in PIV we did not observe any significant change in autoregulation. Previous reports found no change in MCAv and MAP during late phase II (phase IIb) of the VM in the EF and ML phases of the menstrual cycle (Abidi et al., 2017). However, we report small differences in MCAv_mean_ response to the VM when including the LF phase of the menstrual cycle, with differences observed in VM phase IIb and III. The difference appears to be driven largely by changes in SMCAv. Importantly, MAP during the VM and P_ET_CO_2_ post VM were not different between menstrual cycle phases.

### 4.4 Limitations

The use of transcranial Doppler as a proxy for cerebral blood flow is reliant on a stable arterial diameter. During modest changes in MAP, similar to those observed in the VM in the current study (See Figure 4), Giller et al., 1993 reported a <4% change in MCA diameter. Although the role of the sympathetic nervous system in cerebrovascular function is still debated (Ainslie & Brassard, 2014; Brassard, Tymko, et al., 2017), it has been suggested that sympathetic vasoconstriction reduces MCA cross‐sectional area during hand grip by 2% (Verbree et al., 2017). As the VM likely increases cerebral sympathetic activity (Zhang et al., 2004), particularly in the later stages of the maneuver (PIV), constriction of the MCA may occur. There is also some evidence to indicate that muscle sympathetic nerve activity is affected by the menstrual cycle at rest (Carter et al., 2013) and during orthostatic challenges (Carter et al., 2009; Fu et al., 2009) with increased sympathetic activity during the ML phase. Although, if the cerebral sympathetic tone is modified during the menstrual cycle is, to the authors’ knowledge, not known. As the interventions utilized to test cerebral autoregulation in the current study produced modest changes in blood pressure and likely increased sympathetic output to cerebral vessels, MCAv results must be interpreted with caution. With respect to the effect of estrogen on arterial diameter, ICA diameter (Krejza et al., 2001; Nevo et al., 2007), MCA, and basilar artery diameter (Cote et al., 2021) appear stable irrespective of circulating estrogen.

As mentioned previously, few studies have investigated the effect of ovarian hormones on cerebral autoregulation. Our a priori power calculation and data from 12 participants produced correlations for RoR following sit‐to‐stand maneuvers as presented in Figure 2. Whilst the VM data was reduced to *n *= 9, these data are mostly in agreement with previous studies (Favre & Serrador, 2019) with a larger cohort. Nevertheless, further research is required to confirm our findings due to the paucity of data including accurate measurement of ovarian hormone concentration across the menstrual cycle. Additionally, cerebral autoregulation demonstrates hysteresis (Brassard, Ferland‐Dutil, et al., 2017); more effective stabilization of cerebral blood flow during hypertensive compared to hypotensive stimuli. During acute increases in perfusion pressure cerebral vessels constrict to reduce hyperperfusion, although as estrogen dilates small cerebral vessels and modifies myogenic reactivity (Geary et al., 2000), autoregulation during increases in blood pressure may be differentially affected during the LF phase.

### 4.5 Perspectives and significance

The fact that there are differences between men and women in cerebrovascular disease rates (Appelros et al., 2009; Seshadri et al., 2006) indicates that the primary ovarian hormones play a possible role in cerebrovascular function. This is supported by a recent meta‐analysis that showed post‐menopausal women receiving hormone replacement therapy had an improved pulsatility index compared to those not receiving this treatment, and that cerebrovascular reactivity was lower during high than low hormone phases (Skinner et al., 2021). That there is no consensus amongst researchers whether the menstrual cycle should be controlled for in studies assessing vascular function in women (Cohen, 2013; Krejza et al., 2013) is likely one of the contributors to the low reporting (37%) of ovarian hormone concentrations within an already limited literature, alongside methodological differences in assessing cerebrovascular function (Skinner et al., 2021). Whilst we have shown only a small potential influence of estrogen on cerebrovascular function during forced perturbations in blood pressure our data contributes to the growing body of research in this area. These data also support the recommendations by Skinner et al. (Skinner et al., 2021) that future studies including women (e.g., investigating sex differences, pregnancy, menstrual cycle, menopause, hormone replacement therapy, oral contraception, etc.) must report hormone concentrations as standard to highlight individual variability.

## CONFLICTS OF INTEREST

There are no conflicts of interest.

## AUTHOR CONTRIBUTIONS

Stephanie Korad, Toby Mündel and Blake G. Perry were responsible for the study concept and design. Stephanie Korad and Blake G. Perry collected the data. All authors contributed to the analysis of data, drafting this manuscript and all authors approve the final version. All authors agree to be accountable for all aspects of the work in ensuring that questions related to the accuracy or integrity of any part of the work are appropriately investigated and resolved. All persons designated as authors qualify for authorship, and all those who qualify for authorship are listed. Data were collected at the School of Health Sciences, Massey University, Wellington, New Zealand.
